# Striving to recover – wrist splint or plaster cast: a qualitative study of patients’ experience of recovery after a distal radius fracture

**DOI:** 10.1302/2633-1462.66.BJO-2025-0033

**Published:** 2025-06-03

**Authors:** Emma Elizabeth Phelps, Elizabeth Tutton, Jenny Gould, Liz Baird, Juul Achten, Matthew L. Costa

**Affiliations:** 1 Kadoorie, Oxford Trauma and Emergency Care, Nuffield Department of Orthopaedics, Rheumatology and Musculoskeletal Sciences, University of Oxford, Oxford, UK; 2 Major Trauma Centre, Oxford University Hospital NHS Foundation Trust, John Radcliffe Hospital, Oxford, UK; 3 Patient and Public Involvement and Engagement (PPIE) Partner, Oxford, UK

**Keywords:** Qualitative, Interviews, Patient experience, Wrist fracture, wrist splint, plaster cast, splint, wrists, wrist fracture, Randomized Controlled Trials, fractures of the distal radius, strength, distal radius, radiograph

## Abstract

**Aims:**

We sought to explore patients’ experience of early recovery from a fracture of the wrist (distal radius). This study was nested in the DRAFT-CASP randomized controlled trial (RCT), which explores the effectiveness of two treatment pathways for patients with a fracture of the distal radius that does not require manipulation: a plaster cast which is removed in fracture clinic, versus a wrist splint that patients remove themselves without returning to hospital.

**Methods:**

Qualitative interviews were undertaken with 21 adults (mean age 58.2 years (SD 13.96), six male), from eight NHS hospitals, four to ten weeks post injury. Interviews were informed by phenomenology and analyzed using reflexive thematic analysis.

**Results:**

We identified the overarching theme ‘striving to recover’, which conveys patients’ determination to get back to normal after a wrist fracture. To recover, patients needed to be comfortable, to adapt, and to be certain that their wrist was healing. Early in their recovery, they were unable to complete their daily activities, experienced pain, loss of strength, worry, and were cautious about using their wrist. Overall, both treatments were considered acceptable. The splint was advantageous for the freedom and control it provided. The cast was valued for the protection and safety it provided. Both groups required more information and reassurance, but had varied views on the need for follow-up appointments.

**Conclusion:**

The splint made life easier for patients and was an acceptable treatment. Patients wanted reassurance that their wrist was healing, but they felt this could be achieved in a variety of ways. Most patients coped without a follow-up appointment. Innovative ways to maximize recovery are required. These include support for patients to, manage their pain and provide comfort, be able to adapt, and feel certain of healing. Sharing patients’ experiences may help future patients to make informed treatment and recovery decisions.

Cite this article: *Bone Jt Open* 2025;6(6):626–634.

## Introduction

Wrist fractures (fractures of the distal radius) are a common traumatic injury, affecting over 100,000 people in the UK each year.^1^ If the bones at the wrist have moved out of their usual anatomical position, patients may be offered a manipulation of their fracture to restore the anatomy, or even surgery. However, for the majority of patients, where the bones have remained in good alignment, the fracture may be treated with a simple cast or splint. A wrist fracture disrupts patients’ lives and their performance of meaningful activities. Patients experience distress, fear, and loss of control, as well as pain and functional limitations.^2-4^ A lack of information and knowledge about what to expect during recovery leads patients to experience uncertainty about how to use and move their wrist safely.^2,5^

Typically, in the UK, patients with a fracture that does not require manipulation are provided with a temporary backslab (partial plaster) cast in the emergency department (ED) and referred to the fracture clinic.^6^ In the fracture clinic, this cast is converted into a full, circumferential cast. The patient then returns to have their cast removed around four to six weeks later. There is some evidence showing that a removable wrist splint may provide the same support as a cast while the fracture heals. The DRAFT-CASP randomized controlled trial (RCT) will compare the clinical and cost-effectiveness of a removable wrist splint compared with a plaster cast for distal radius fractures that do not require a manipulation. Patients who take part in the RCT and are randomized to a splint will be discharged directly from the ED, as they can remove the splint themselves. Patients who are randomized to a cast will have at least one follow-up appointment to remove the cast. In both treatment groups, patients will receive a detailed rehabilitation booklet which includes instructions for strengthening exercises, information about recovery from a wrist fracture, and when to seek help.^6^ Little is known about patients’ experience of a removable wrist splint after a wrist fracture and how patients manage their recovery when discharged directly from ED. This qualitative study therefore examines and compares patients’ experiences of both treatments used to treat a distal radius fracture in the DRAFT-CASP RCT.

## Methods

### Participants

A purposive sample of 21 patients (six male) from eight NHS hospitals across the UK, participating in the DRAFT-CASP study, took part in a telephone interview. A total of 12 patients had been randomized to a removable splint; eight were discharged from the emergency department without a follow-up appointment. Of the nine patients who received a cast, three changed to a splint within two weeks of their injury and two were given a splint after their cast was removed at four to six weeks post injury. Participants were aged between 37 and 84 years (mean age 58.2 years (SD 13.96)). Interviews took place from June 2023 to January 2024. Participants were interviewed between 30 and 67 days post injury (mean 52.67 days (SD 9.91)).

### Interviews

The interviews drew upon Heideggerian phenomenology and notions of Dasein (‘being there’ or ‘presence’).^7^ This methodology allows us to explore the ‘lifeworld’ of participants, and has been useful in other studies of injury in adults.^8,9^

Patients were informed about the qualitative study as part of the initial consent process for the DRAFT-CASP study. Those who were interested in taking part in an interview provided electronic consent to be contacted. They were emailed an information sheet about the qualitative study and given the opportunity to ask questions. Patients who agreed to take part underwent a separate informed consent discussion, with verbal informed consent recorded and witnessed by an administrator who had undertaken research integrity training referred to as good clinical practice (GCP).

Interviews, conducted by an experienced female qualitative researcher (EEP), were semi-structured and enabled participants to say what was most important to them; they lasted up to 50 minutes. Interviews explored patients’ experience of injury, treatment, recovery, their experience of being asked to take part in the DRAFT-CASP study, and their views on the treatment pathways.

Interviews were audio-recorded, transcribed, and managed using NVIVO 12 (QRS, UK), and field notes were written after each interview. Data were analyzed inductively using a reflexive approach to thematic analysis.^10^ EEP listened to the recordings, read the transcripts, and wrote field notes to gain an understanding of the important elements of each participant’s experience. Data were coded line by line, with codes first grouped to form categories and then into themes. Analysis was iterative, and throughout analysis EEP and ET discussed and reflected upon the data and developing themes. Further discussions took place with the co-authors of this article.

Rigour and trustworthiness were achieved by familiarization with the data and ongoing reflection and discussion throughout the analysis.^11^ To enable readers to consider the transferability of these findings to other contexts, detailed descriptions of the context and methods are provided and quotations are included to illustrate our interpretations of the data. The consolidated criteria for reporting qualitative research (COREQ) guidelines informed this article.^12^

### Patient and Public Involvement and Engagement

Patient and Public Involvement and Engagement (PPIE partners have been involved in the DRAFT-CASP study and this qualitative study by contributing to: 1) the study design; 2) development of study materials; 3) the DRAFT-CASP study management and steering committees; 4) discussion of the qualitative findings; and 5) the development of this article.

### Ethical approval

The DRAFT-CASP study is registered with the International Standard Randomized Controlled Trials Number Registry (ISRCTN66692543: 27 January 2023). The South West - Frenchay Research Ethics Committee approved the DRAFT-CASP study, and this embedded qualitative study (REC reference 22/SW/0177).

## Results

**Fig. 1:**
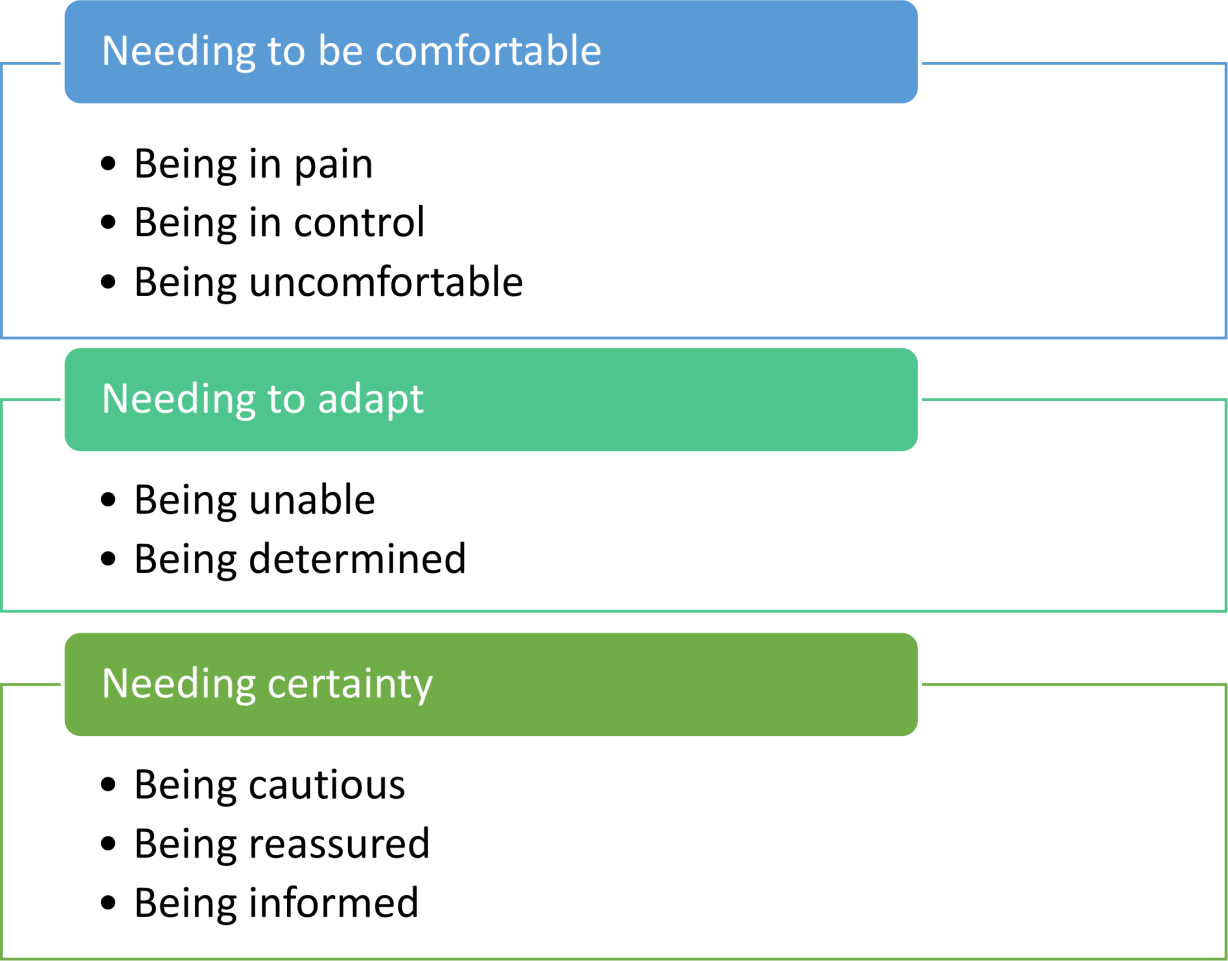
Striving to recover: themes and categories.

The overarching theme of ‘striving to recover’, identified in this study, conveys patients’ efforts and determination to get back to normal after a wrist fracture. This is depicted through three themes (Figure 1), which show that in order to recover, patients need to be comfortable, to adapt, and to have certainty that their wrist is healing as expected.

### Theme 1: Needing to be comfortable

Being in pain: Patients experienced pain, stiffness, and loss of strength, which were often worse and lasted for longer than expected. Pain, stiffness, and lack of strength hindered patients’ ability to complete everyday tasks such as picking up children and opening jars. Several patients found that placing their wrist in warm water provided relief from their pain.

“The A&E doctor said I’ll warn you now, whether you have a cast on or a splint on, it’s going to be very painful. I said, okay, fair enough and true enough in the first four weeks I thought she was right about that, it was bloody agony.” (Patient 15, cast-splint)

By the time of their interview, pain had subsided for most patients but was still experienced with certain movements. Patients tried to improve their strength and prevent stiffness by keeping their wrists moving. Some patients felt they did too much as their wrist became sore.

“As I say it’s only certain movements, I can feel just up the side of the wrist a little bit, but that’s it. I mean it’s not even a pain really, it’s like a pull.” (Patient 19, splint)

Being in control: A splint gave patients a degree of control and freedom. They could loosen or tighten the splint to their own comfort, remove the splint, and move their hand and fingers. They could wear clothes with sleeves, and patients with a spare splint could wear one while washing the other. Three patients who lived alone and were allocated a cast changed to a splint as they found the cast too restrictive.

“As the swelling went down, which took a long time, I could then tighten it. I could do it to my comfort, I could loosen it or tighten it.” (Patient 13, splint)

Although patients wanted the option to remove the splint, they often found this was too painful during the early phase of their recovery. Instead, patients undid the splint to rest, wash, scratch, or put cream on their wrist, and some bought covers so they could shower with the splint on.

Patients were advised to remove their splint four to six weeks after injury. Once removed, patients only put their splint back on when they felt they needed it – for example, when going outside, to crowded places, as a reminder not to use their wrist too much, while sleeping, or as a visible sign of injury for others. While the splint was protective and reassuring for many patients, a minority felt reliant on the splint.

Conversely, some patients in a cast were relieved that they could not be tempted to take it off in the heat, found the cast comfortable and felt their wrist was protected.

“I felt like the cast was protecting it and keeping it very much in place.” (Patient 20, cast-splint)

Being uncomfortable: Patients in both treatment groups experienced discomfort. The cast could be itchy, could rub on the skin, and some patients experienced pain around the thumb where the cast felt too tight. Some patients found it challenging to sleep comfortably with the cast and the cast impacted upon their hygiene. Patients who received a splint described a cast as miserable, uncomfortable, smelly, itchy, and difficult to sleep with, based on their own past experiences or experiences of others.

A minority found the splint uncomfortable or felt that their wrist was unsupported. The flaps on the splint could be sharp and dig into the skin. The splint could also feel hot and sweaty, and it could smell; many patients wanted two of them so that they could alternate.

“The splint is a bit awkward. It’s got these funny little straps on, there are three little straps that are as sharp as can be.” (Patient 1, splint)

Table I provides illustrative quotations for the theme of needing to be comfortable.

**Table I.** Needing to be comfortable: illustrative quotations. Codes are provided after the patient number and treatment group.

Theme 1: Needing to be comfortable conveys patients’ struggle with pain, stiffness, and loss of strength; the value of being able to remove and adjust the splint themselves; and the discomfort caused by the cast/splint.	
Being in pain conveys patients’ struggle with pain, stiffness, and loss of strength, which was often more severe and lasted longer than expected.	“I tried to sit still just to make sure I didn’t do anything stupid but, yes, it was just a little bit weak I think is the word I would say to describe it. It was a bit like a ‘jelly’ almost, like it didn’t quite feel like it was set properly.” (Patient 20, cast-splint) ‘It was a bit like a jelly’ “Well, it’s more around the lack of strength, there’s absolutely no strength in it and so it’s really hard to open jars and stuff like that which is a kind of given. You don’t put a second thought to that stuff normally, so at the minute it’s just very weak.” (Patient 5, splint) ‘It is very weak’ “I think the amount of pain I had with it surprised me… They say hairline and you think something really quite simple don’t you… That’s the only thing I would go back for [i.e. get advice], how much pain I do still have in it. It does restrict me without a doubt just getting dressed, taking your underwear off. I have to think about which way I’m turning it [wrist] and, yes, it’s just on that rotation, it’s just catching it.” (Patient 13, splint) ‘It does restrict me’ “Still now I will suddenly roll over and pull up my duvet or something and that wakes me up because it hurts. There’s still pain there.” (Patient 10, Cast) ‘It wakes me up’
Being in control conveys the value patients placed on being able to remove and adjust the splint to their own comfort.	“I’ve found because the splint was a little bit more flexible you could sort of gently move it [wrist] around in the splint, you just had a little bit more movement so it wasn’t quite as uncomfortable as a cast can get. I’d certainly recommend it if anyone was given that choice and they were in the same position as me.” (Patient 15, cast-splint) ‘A little bit more flexible’ “I took it off after six weeks when I was indoors and when I was at home, I was quite comfortable with it but if I went into town or had to go to the shops, I had it on because I wasn’t comfortable with people pushing past me and rushing into me.” (Patient 12, splint) ‘Protecting myself’ “Yes, very secure but when I took it off, I felt a bit - it felt funny when I first took it off. I felt as though I couldn’t wait to get it back on. I didn’t but I knew I could have done. I know I could have put that back on at any time, but I didn’t, and I was fine.” (Patient 3, Splint) ‘I couldn’t wait to get it back on’ “I like the splint, I like the ability if I wanted to loosen it, tighten it, take it off, if necessary. I quite like that, and I like the control, I think most people do, you want control over your own body as long as you think you’re not doing yourself harm - there’s no detrimental impact in doing it.” (Patient 5, splint) ‘Control over your body’ “We had a trip down to London and I’d had it on for two weeks by that point and we went across the underground and it was really, really hot and sweaty and I remember thinking, oh my god, I just really want to take this off. So it was good that I didn’t have the option of taking it off.” (Patient 4, cast) ‘Good I was not able to take it off’ “Although it’s easier in a splint because you can take it off, if need be, but you don’t feel quite so secure mentally as you do when you’re in plaster. You know it’s not going to move when it’s in plaster.” (Patient 16, cast) ‘Protected in plaster’
Being uncomfortable conveys the discomfort patients experienced while wearing their cast or splint.	“I was really itchy and when they took it off there was a bit underneath of my skin that had obviously reacted to something, I don’t know. I got on alright, I just lived with it.” (Patient 10, cast) ‘Skin reaction’ “It didn’t help that a part of the cast was digging into my thumb and so I’ve got a really bad sore where my thumb is and that’s constantly in pain at the moment.” (Patient 9, cast) ‘Sore on thumb painful’ “It [the splint] was awful, and it didn’t feel like it gave you any support whatsoever. By the time I came home my hand had swollen half the size again. The [local] hospital had said to me if you find the splint that they gave you in [the first] hospital more comfortable then I could wear that because it will do exactly the same. [The first] hospital was pretty good because they measured my arm and so they got me a splint that really fitted me very well.” (Patient 7, splint) ‘Being measured to fit’

### Theme 2: Needing to adapt

Being unable: A broken wrist impacted upon patients’ day-to-day life. Washing, toileting, getting dressed, and brushing teeth were challenging during the early weeks. Patients struggled to cut food, open jars, and lift things. They needed help from other people including family members, or adapted to using one hand. Movements which had previously been taken for granted now had to be thought about, for example which way to turn their hand when dressing, or testing the weight of shopping bags before lifting them.

“I couldn’t wash my hair, I couldn’t drive, I couldn’t dress properly, and it was definitely an impediment.” (Patient 17, splint)

Patients in manual jobs were unable to work after their injury. Office-based jobs were also difficult, as typing and using a computer mouse were challenging. Patients could not drive in the first weeks after injury, impacting upon work for some. Parents or grandparents of young children could struggle to lift them, help with childcare, or change nappies. Patients’ sleep could be affected by their pain: some struggled to position their arm comfortably, while others woke up in pain from moving in their sleep.

At the time of their interview, some patients were back to doing almost everything they had been doing prior to their injury. Other patients still struggled with some tasks including opening jars and wine bottles, cutting food, and lifting milk bottles, shopping bags, children, and/or pets. Some patients who had resumed driving still did not feel completely confident doing so, or found it hard to use the handbrake. Patients who had returned to the gym were often not able to lift the same weight as before.

Being determined: Patients were determined to keep going and get back to normal. They did not want to feel they were giving up. Patients learnt to adapt and find alternative ways of doing things: “if something needs doing, I will find a way to do it.” Many tasks usually requiring two hands were done one-handed, such as getting shampoo out of the bottle. Patients who broke their dominant wrist found that they could learn to do more than expected with their other hand.

Most patients tried exercises to help their recovery, including moving fingers and squeezing socks or a ball. Some patients could do all the exercises in the exercise booklet they were given, while others found them difficult due to stiffness, or would have already struggled to do them before their injury. Patients often described moving their wrist or fingers while resting, even if they forgot to follow the recommended exercises.

“I started doing the exercises as soon as I got home really because I thought I need my arm back.” (Patient 19, splint)

At times, some patients felt they had overdone it, and experienced pain. Some patients needed to make themselves use their wrist again, trying to motivate or push themselves to get back to how they were.

Illustrative quotations for the theme of needing to adapt are presented in Table II.

**Table II.** Needing to adapt: illustrative quotations. Codes are provided after the patient number and treatment group.

Theme 2: Needing to adapt conveys patients’ struggle with their day-to-day activities, and their determination to keep going and use their wrist again.	
Being unable conveys the impact of injury on patients’ day to day life and their struggle to complete everyday tasks.	“I think the combination of the wrist and COVID made me feel very old and I think that’s one of the reasons it took me a while to bounce back from it, because I was really quite, well, not depressed but it made me feel quite vulnerable. Vulnerable is a good word actually.” (Patient 4, cast) ‘It made me feel vulnerable’ “I still struggle getting dressed and doing hygiene in the shower and stuff like that but that’s because my arm has not had physio yet. So, my muscles are still very tight.” (Patient 2, splint) ‘Struggle to dress and shower’ “I’m driving again but I do find it difficult if I have to try and pull up the handbrake, that’s when I feel it or if I try and grip or lift. If I try to add milk to my coffee with my left hand with a four-pint carton I suddenly feel I can’t do that. There are just some actions and it’s not so much lifting, I can lift, but it’s the actions and it’s not the bone, it’s the muscles and tendons, from being immobilized I guess rather than from the bone injury.” (Patient 10, cast) ‘I can’t grip and lift’ “It’s the mouse and computer. I’ve been trying to use my laptop and mouse, and I can do it for a certain amount of time. I’d probably say within an hour I’m probably in pain and have to stop. Of course, it’s a seven-and-a-half-hour day, but hopefully I’m doing a phased return, which I think is probably going to be the best thing for me, because I don’t want to be pushing it and then get signed off again because I’m in absolute agony.” (Patient 9, cast) ‘I can do it for a while’
Being determined conveys patients’ efforts to find new ways of completing tasks and their desire to get back to their pre-injury normal.	“I just did everything one-handed, so I did manage to hoover and do the lawn and just do things one-handed.” (Patient 10, cast) ‘Did everything one-handed’ “I wanted to take it off, but I didn’t want to use it too much and even just things like getting the shampoo out of the bottle. How you squeeze it out with your left hand because I never put it straight onto my head. Things like that and it’s just working out different ways of doing things.” (Patient 13, splint) ‘Different ways of doing things’ “I think I learned to use my left hand very, very well. It was the simple things that everybody takes for granted really and especially because it was my right hand, I couldn’t wash my hair, couldn’t dry it but the girls did that. I love gardening but I couldn’t use the secateurs because they don’t work on your left hand when you’re used to your right, funny things like that really but I just either didn’t do them or learnt to use my left hand. You actually don’t realise how much you use your left hand anyway when your right hand is dominant. When you haven’t got that right hand anymore, you realise that actually you use your left hand more than you ever realise you do.” (Patient 7, splint) ‘Using my left hand’ “So, I’ve just adapted really. I’m a tough old bird and I’m not going to let anything – you know I wouldn’t just sit and give up and so I did most things. I still lifted the shopping, but I did it with my left hand instead of my right. The only thing that I really couldn’t do because it was uncomfortable was hold the baby.” (Patient 7, splint) ‘Not giving up’

### Theme 3: Needing certainty

Being cautious: Patients worried about their recovery. They were cautious about using their wrist and tried to protect it, as they feared further injuring their wrist or hindering their recovery. Patients tested what they could do without pain, testing the weight of items before lifting them. They were also careful not to fall again.

“I almost felt that I was a little bit scared of what I might do to it. It sounds ridiculous doesn’t it, but I just kept thinking should I be doing this/should I be doing that.” (Patient 13, splint)

Some patients worried about the levels of pain they experienced, felt that their joints were moving, or were worried about their lack of strength. They questioned whether they would get back to how they were before.

Being reassured: Patients wanted certainty that their wrist was healing as expected, but had different views on the importance of follow-up appointments. Patients who had a radiograph were reassured to know that their wrist was ‘knitting together’, while some patients who had an appointment without a radiograph often wanted one to be certain that their wrist was healing. Some patients who had face-to-face follow-up appointments felt that they would have been okay with a telephone call or detailed information addressing their concerns with contact details for further support. Others questioned whether they would feel unsure about whether their wrist was healing if they did not have the appointment.

“I think I would certainly be happier knowing, just to have an x-ray and to have it checked over to make sure that everything was as it should be.” (Patient 18, cast)

Patients who did not have follow-up appointments often questioned whether their wrist was healing, though they often believed that if there was a problem there would be a sign or symptom. Several patients planned to get their wrist checked if the pain they were experiencing at the time of their interview continued. Others who had considered contacting a healthcare professional about their wrist found that their pain improved, so they no longer needed to do so. Patients who planned to get their wrist checked often intended to visit their GP rather than contact the fracture clinic or return to hospital.

Long waiting times, little information and reassurance during follow-up appointments, and difficulty parking or getting to hospitals led some patients to prefer not to have a follow-up appointment.

Being informed: Patients spoke of the support they received from clinical staff. They were reassured by staff answering questions and providing information about recovery. Staff gave hope that patients’ wrists would recover with time. A minority of patients commented on how quickly they were given a splint and discharged: they felt “kicked out the door” (Patient 1, splint) and as though staff were trying to get rid of them. Sometimes they felt that they did not have enough information about their fracture and expected recovery. One patient questioned whether the time to apply a cast would allow more time for staff to give information to patients and talk to them about what to expect during their recovery.

Illustrative quotations for the theme of needing certainty are presented in Table III.

**Table III.** Needing certainty: illustrative quotations. Codes are provided after the patient number and treatment group.

Theme 3: Needing certainty conveys patients’ worry about the uncertainty of their recovery which led them to be cautious with their wrist; want reassurance their wrist was healing; and want more information about recovery.	
Being cautious conveys patients' hesitancy to use their wrist and need to keep their wrist protected.	“When you try to do it [lifting] you just test it, like boxes and things like that. Is it going to be light or heavy and if it’s too heavy, I don’t lift it. I will just see how heavy it is to see if I can manage. It’s that sort of thing but I think that caution of mine will probably pass in the next week or two because it’s quite new. It’s only a couple of weeks [after removing the splint] and quite early really.” (Patient 12, splint) ‘Cautious when lifting’ “I’m not quite sure how I’m going to go about using them [tools for work]. As I say the way it looks today, I should be testing it very gingerly next week and then seeing how far I get with it. I suspect there will be restrictions, but I don’t think it’s going to be long term.” (Patient 1, splint) ‘Am I ready to work’ “Because of the pain my mind is telling me to be concerned about pulling it apart. So, I’m giving myself plenty of time. Being able to pick up a suitcase or something would be frightening if it wasn’t clear in my head that it was repaired properly.” (Patient 16, cast) ‘Giving myself time to know my wrist is repaired’
Being reassured conveys patients’ need for confirmation that their wrist is healing as expected.	“I think the thing is with not having to go back to the hospital, I was thinking I hope it has mended okay. I’ve got no problems with it. That is the only thing with not having the cast, with not seeing so many medics to say, yes, you’re fine but I think I’d be having a lot of problems if it had not mended properly.” (Patient 19, splint) ‘I hope it has mended’ “In my mind no-one’s ever seen my x-ray that shows it’s repaired, so that’s a little bit of fear in me that I don’t like.” (Patient 16, cast) ‘Confirming healing via an x-ray’ “I think I would certainly be happier knowing, just to have an x-ray and to have it checked over to make sure that everything was as it should be. Yes, because although if they’d said it had healed, I think I would still be concerned at using my wrist… I go to a gym and part of the gym work we do is lifting weights and using wrists an awful lot. I am concerned about doing that. So again, I will ask the doctor whether he thinks I should wait a while because I don’t know how easy it is, once it’s been broken, for the same thing to happen again.” (Patient 17, splint) ‘Concerned about lifting weights’ “It kind of felt like it [follow-up appointment] tied it up a bit. So, I knew what to go and do from there. So, it was quite nice to have that contact, I can’t lie, but at the same time I probably would have survived without that contact. If I was given information somewhere online where I could go and look at it and it said all the things they told me. Then I would quite happily have taken that information in that kind of way as well.” (Patient 20, cast-splint) ‘Knowing what to do’ “I think if this pain continues, as it is, then maybe in another three or four weeks, I think then I would just get somebody to maybe check it out.” (Patient 13, splint) ‘Check wrist healing due to pain’
Being informed conveys patients’ need for information to support their recovery.	“He was very reassuring, and he asked me some questions and one of them was ‘can you push yourself up with that hand?’ and I said ‘no’, and he said, ‘not yet’ and I thought well there’s hope there.” (Patient 4, cast) ‘There is hope’ “If they hadn’t just put the splint on me and kicked me out the door, I think I would have gone home a little bit happier… If they had walked me through it a bit more, I think I would have been a bit more relaxed. She had two of these straps and stuck it on and kicked me out the door. If you go down to get a plaster of Paris put on, they take their time with you. So, they relax you and get you accustomed to whatever it is, and so you go out with a better feeling.” (Patient 1, splint) ‘Felt kicked out the door’ “The advice at the time is that you will take about four to six weeks. Then you’re automatically benchmarking against that and then when it wasn’t right. It’s not 100% now but at the time you’re thinking six weeks, and it’s still pretty much splinted up and it’s painful. I can’t do that and you’re thinking why isn’t it healing properly. So, then you start googling and then that’s when you can go down a rabbit hole.” (Patient 5, Splint) ‘Using the internet to find out what to expect’ “I said after three weeks ‘can you give me an idea of how long it’s going to be before I get the full dexterity of it back and the full lifting capacity of it’. She said that was going to be twelve weeks. I said, ‘twelve weeks from the start,’ and she said ‘no, another twelve weeks,’ I thought crikey that’s a long time isn’t it.” (Patient 15, splint) ‘A long time to recover fully’ “It was quite nice to be able to ask some specific questions about me. Although there’s probably other people who want to know if they can go back in the gym and so I’m not sure how unique the questions would have been in the bigger picture.” (Patient 20, cast-splint) ‘Specific questions about me*’*

### Comparison of splint and cast

Patients considered the removable splint an acceptable alternative to a cast, and often took part in the DRAFT-CASP trial in the hope of being randomized to a splint. Table IV compares the benefits and disadvantages of a splint and plaster cast, as described by our participants.

**Table IV.** Comparison of splint and cast.

	Splint	Cast
Advantages	**+** Can remove to wash, scratch, apply cream, and rest **+** Can tighten and adjust to own comfort **+** Can replace and wash the splint if you have two **+** Can fit through sleeves so don’t need to cut clothes **+** Can put it back on for protection if going outside, or while sleeping **+** Able to use fingers **+** Less restrictive	**+** Protection **+** Security as others can see you are injured **+** No temptation to remove **+** The follow-up appointments associated with treatment in a cast can provide support and reassurance **+** Patients who had a radiograph could feel certain their wrist was healing as expected
Disadvantages	**-** Can be uncomfortable if it doesn’t fit well **-** Hot, sweaty and smelly **-** Can feel reliance on the splint **-** Can feel unsupported **-** No/less follow-up **-** Feeling less informed, kicked out **-** Uncertain about recovery	**-** Hot, sweaty, and smelly **-** Itchy **-** Can cause soreness/rubbing **-** Often need to cut up clothes **-** Restrictive **-** Difficult to sleep and wash **-** Travel, parking, and time to attend and wait for follow-up appointments can be inconvenient

## Discussion

This study identified the theme of ‘striving to recover’, reflecting patients’ determination to get back to normal after a wrist fracture and return to activities that were important to their life and identity. The removable wrist splint was an acceptable alternative to a cast, and patients valued the greater freedom and control the splint afforded. The thinness of the splint, ability to change the tightness, greater use of their fingers, and access for skin care were valued. Both splint and cast provided protection, but the cast was thought to provide more protection in early recovery. Pain limited splint removal and contributed to patients’ feelings of caution when using their wrist.

Patients felt that they needed more information and support, which has been identified in several previous research projects involving patients with a fracture of the distal radius.^2-5^ They wanted to know what to expect during their recovery, reassurance that their experience and particularly their pain was normal, and certainty that their wrist was healing. Our findings indicate that the quality of follow-up appointments was inconsistent, and most patients who did not have a follow-up appointment coped despite initially wanting their wrist to be seen. Patients needed to feel reassured that their wrist was healing as expected, but felt that this could be achieved in a variety of ways. For some patients, a radiograph to confirm healing was reassuring, others wanted to speak to a healthcare professional either in person or by telephone, while some felt that receiving information at discharge, including when and how to seek further help, would be sufficient. Information tailored to their own circumstances was important to some patients.

Sharing patient experiences may help future patients to make sense of their recovery. Stern et al^3^ found that patients were not able to understand recovery until they had experienced it, which may in part explain why patients felt uninformed, even though they had received written information about recovery and rehabilitation as part of the DRAFT-CASP trial. Patient activation, that is, a person’s knowledge, skill, and confidence to manage their own health and wellbeing, is associated with lower disability and higher satisfaction, and may be important for recovery after injury.^13^ Our findings show that patients wanted to know what level of pain is expected and when to seek help. Further research is needed to understand when and how to deliver support and information to patients in an accessible way to encourage patient activation and maximize recovery, and how to provide reassurance to patients without resorting to a radiograph.

This study used a purposeful sample of patients from across the UK, including patients who received both study interventions, and three patients who changed intervention within two weeks of randomization. We included a range of ages and sex to capture the breadth of experience. However, we have no evidence that the sample was ethnically diverse or included participants from a range of socioeconomic backgrounds. All participants in this qualitative study took part in the DRAFT-CASP RCT – patients who declined to take part may have had different experiences, views, and concerns.

In conclusion, in ‘striving to recover’ after a broken wrist, patients endeavoured to keep going but needed comfort, to adapt, and reassurance that their wrist was healing. The removable splint was an acceptable alternative to a plaster cast and made life easier. Patients in both treatment groups expressed a need for more information, reassurance, and support to ease their feelings of uncertainty, caution, and worry. Innovative ways to support patients’ determination to recover are required to maximize their recovery potential. Further research is needed to identify the optimal way to support patients after a wrist fracture. Sharing patient experience may help future patients to make informed treatment and recovery decisions.

**Take home message**

- Wrist fractures have a major impact on patients’ daily life. In the first few weeks after their injury, they experience pain, loss of mobility and strength, feel worried, and are cautious of using their wrist.

- Patients find ways to manage pain, cope with discomfort (e.g. itchiness, sweatiness, loss of control), and adapt, but would value further support and reassurance about fracture healing.

- Further research is needed to identify the optimal way to support and reassure patients.

## Data Availability

The data that support the findings for this study are available to other researchers from the corresponding author upon reasonable request.
